# Epidemiology and clinical management of elbow joint disease in dogs under primary veterinary care in the UK

**DOI:** 10.1186/s40575-020-0080-5

**Published:** 2020-02-14

**Authors:** Dan G. O’Neill, Dave C. Brodbelt, Rebecca Hodge, David B. Church, Richard L. Meeson

**Affiliations:** 1 Production and Population Health, The Royal Veterinary College, Hawkshead Lane, North Mymms, Hatfield, Herts AL9 7TA UK; 2 Clinical Sciences and Services, The Royal Veterinary College, Hawkshead Lane, North Mymms, Hatfield, Herts AL9 7TA UK

**Keywords:** General practice, First opinion, VetCompass, Arthritis, Degenerative joint disease, Osteoarthritis, Elbow dysplasia

## Abstract

**Background:**

Conditions affecting the elbow joint are a common cause of lameness in dogs. Primary-care veterinary clinical data are now recognised as a valuable research resource. Using data from the VetCompass Programme, this study aimed to report the frequency and risk factors for elbow joint disease in dogs under primary veterinary care in the UK and describe clinical management.

**Results:**

From 455,069 dogs under veterinary care, the one-year period prevalence for elbow joint disease diagnosis was 0.56% (95% CI: 0.53–0.60). Of 616 incident cases, the most common specific variants of elbow joint disease were osteoarthritis (*n* = 468, 75.97%), elbow dysplasia (190, 30.84%) and traumatic (41, 6.66%). The most common signs described by the owners were lameness (*n* = 466, 75.65%), difficulty exercising (123, 19.97%) and pain (86, 13.96%). The most common findings recorded on veterinary examination were pain (*n* = 283, 45.94%), lameness (278, 45.13%) and reduced range of movement (243, 39.45%). Common medications used included non-steroidal anti-inflammatory drugs (*n* = 544, 88.31%), tramadol (121, 19.64%) and disease modifying agents (118, 19.16%). Of 109 deaths involving euthanasia with information available from the 616 incident cases, elbow joint disease contributed to the decision to euthanase in 45 (41.28%) dogs.

Five breeds showed increased odds of elbow joint disease compared with crossbred dogs: Rottweiler (OR: 6.16, 95% CI 3.89–9.75), Labrador Retriever (OR: 5.94, 95% CI 4.65–7.60), German Shepherd Dog (OR: 4.13, 95% CI 2.88–5.93), Golden Retriever (OR: 3.11, 95% CI 1.93–5.00) and English Springer Spaniel (OR: 2.00, 95% CI 1.26–3.18). Additional risk factors included having an adult bodyweight that was equal or higher than their breed/sex mean, advancing age, being male, being neutered, being insured and larger bodyweight.

**Conclusions:**

Elbow joint disease is a relatively common diagnosis in dogs and has a high welfare impact as evidenced by the high proportion of cases recorded with pain, lameness and analgesic therapy. There are strong breed predispositions, in particular for large breed dogs. These findings present a clear case for improved breeding programmes to reduce the burden of elbow joint disease.

## Plain English summary

The canine elbow can be affected by several different diseases (e.g. elbow dysplasia, osteoarthritis) that cause pain and loss of freedom to exercise. First opinion veterinary clinical records are a valuable research resource that benefit from recording of medical records at the time of the clinical event. This study aimed to report the frequency and risk factors for elbow joint disease and describe clinical management in dogs attending first opinion veterinary practices in the UK.

The VetCompass™ Programme shares anonymised clinical records with first-opinion veterinary practices in the UK for research. These records on dogs were searched for cases of elbow joint disease and additional information was extracted on each case. The study included 455,069 dogs at 304 clinics in the UK during 2013. The overall proportion of dogs with elbow joint disease was 0.56%. The average adult bodyweight of cases was 32.45 kg and the average age at diagnosis was 6.84 years. The most common presenting signs recorded by owners were lameness (75.65%), difficulty exercising (19.97%) and pain (13.96%).

Five breeds had increased odds of elbow joint disease compared with crossbred dogs: Rottweiler (× 6.16), Labrador Retriever (× 5.94), German Shepherd Dog (x: 4.13), Golden Retriever (× 3.11) and English Springer Spaniel (× 2.00). Dogs that were heavier than their breed/sex had 2.00 times the odds of elbow joint disease. Aging, heavier bodyweight, being male, being neutered and being insured were also associated with increased odds.

At least one medication was prescribed for 93.02% of cases. Common medications used included non-steroidal anti-inflammatory drugs [NSAIDS] (88.31%), tramadol (19.64%) and disease modifying agents (19.16%). Of 109 dogs that were euthanased for any reason during the study period, elbow joint disease contributed to the decision to euthanase in 45 (41.28%).

This study shows that elbow joint disease is a relatively common diagnosis in dogs and has a high welfare impact. There are strong breed predispositions, in particular for large breed dogs. These findings present a clear case for improved breeding programmes to reduce the burden of elbow joint disease in dogs.

## Background

Conditions affecting the elbow joint are a common cause of lameness in both young [1] and older dogs [2]. The elbow was identified as the most frequently abnormal joint in a study of radiographic abnormalities based on screening of 1018 Labradors over one year of age [3]. The canine elbow can be affected by several different diseases, including elbow dysplasia [4], osteoarthritis, humeral intracondylar fissures [5, 6], congenital luxations, soft-tissue problems [7] and septic arthritis [2]. However, each of these diseases usually results in a similar clinical presentation that includes lameness, joint pain and reduced elbow movement, and that adversely affect welfare [1, 7]. Overall, the syndromic group of diseases known collectively as ‘elbow dysplasia’ predominates as the most commonly reported group of conditions affecting the elbow of large and giant breed dogs and generally results in secondary osteoarthritis [1, 4]. The vast majority of literature associated with diseases of the canine elbow focuses on elbow dysplasia and its management, and these studies have generally been based on the referral or disease-screening subsets of the dog population [4, 8].

Elbow dysplasia describes a group of conditions that were first described in 1965 as generalised elbow osteoarthrosis with or without ununited anconeal process and which are thought to arise from abnormal growth of the elbow joint [9]. The International Elbow Working Group (IEWG) publishes an annual report on the state of diagnosis and management of elbow dysplasia in dogs. Two decades after their first landmark report, there remains disagreement about exactly which pathologies of the elbow are forms of elbow dysplasia with variable inclusion of ununited medial epicondyle and elbow incongruity [8, 10]. The undisputed pathologies under this syndromic umbrella term include fragmented medial coronoid process, osteochondrosis of the humeral condyle, and ununited anconeal process [4, 11, 12]. Although ununited anconeal process and osteochondritis dissecans of the humerus are relatively straightforward to identify on plain radiographs, diagnosis of disease associated with the medial coronoid process (including the aforementioned fragmentation) is problematic due to superimposition of the medial epicondyle and other soft-tissues [11–13]. Therefore, diagnosis is based on associated radiographic secondary changes in the joint (ulna notch sclerosis, new bone formation on the anconeal process or radial head) in the absence of radiographically identifiable lesions, as surrogate marker for medial coronoid disease [14, 15]. However, these changes are only indicative of elbow osteoarthritis [16]. Advanced modalities, such as computed tomography and arthroscopy have significantly increased the accuracy of diagnosis of elbow pathologies such as medial coronoid disease [17, 18] but are typically only available in referral clinics and hence only for a small and biased subset of dogs [19].

Elbow dysplasia has a reported prevalence of 17% in US Labrador Retrievers, and 70% in Bernese Mountain Dogs from the Netherlands [20]. Although large breed dogs and males are over-represented [21], increased risk has also been reported in some chondrodystrophic breeds such as the French Bulldog and Dachshund [4]. To date, most epidemiological studies of elbow dysplasia, or elbow disease more generally, have been based on subsets of animals from treated populations (usually referral cases [4, 8, 12]) or disease screening programmes [22–26]. These approaches, however, fail to account for the impact of the condition in the wider general dog population.

Several large epidemiology studies have reported genetic predisposition to elbow dysplasia in certain dog breeds, in particular Rottweilers, German Shepherd Dogs, Bernese Mountain Dogs and Labrador Retrievers, with estimates of heritability ranging from 0.10–0.38 [22–25]. When considering specific diseases such as medial coronoid process disease, there is increased heritability such as in the German Shepherd Dog at 0.57 [26], indicating an important genetic contribution to elbow dysplasia. A bimodal pattern of presentation with two age related peaks has been described for elbow dysplasia; young dogs less than or around 4-12 months of age, and then older dogs of around 8 years [1, 4]. However, these data are based on referral populations where biases such as financial factors may have influenced the patterns seen.

Primary-care veterinary clinical data are now recognised as a valuable research resource that benefit from contemporaneous recording of medical records at the time of the clinical event, and from the recording of cohort data over time and at a veterinary level of clinical precision [27, 28]. Such data have been validated for research purposes by several previous reports on diverse conditions in dogs including road traffic accidents [29], appendicular osteoarthritis [30], dystocia [31], urinary incontinence [32], and corneal ulcerative disease [33]. The current study aimed to fill the information gap on the epidemiology of elbow joint disease by estimating the prevalence and incidence of elbow joint disease in dogs attending primary-care veterinary practice in the UK and evaluating breed as a risk factor for incident elbow joint disease. The study also aimed to report summary statistics on diagnostics, management and outcomes that can contribute to benchmarking for clinical audit and governance [34, 35].

Based on the prior but potentially biased information in the literature, it was hypothesized that purebred dogs, in particular Labrador Retriever, Bernese Mountain Dog, Rottweiler German Shepherds, English Springer Spaniels and French Bulldogs, older dogs, male dogs, and heavier dogs would have higher odds of elbow joint disease than crossbred, younger, female and lighter dogs respectively.

## Methods

The VetCompass™ Programme collates de-identified electronic patient record (EPR) data from primary-care veterinary practices in the UK for epidemiological research [27, 36]. VetCompass™ collects information fields that include species, breed, date of birth, sex, neuter status, insurance status and bodyweight, and clinical information from free-form text clinical notes and summary diagnosis terms (VeNom codes) [37], plus treatment and deceased status with relevant dates. The EPR data were extracted from practice management systems using integrated clinical queries and uploaded to a secure VetCompass™ structured query language database [27].

A cohort study design was used to estimate the prevalence, incidence and risk factors for elbow joint disease [38]. The sampling frame for the current study included dogs under veterinary care within the VetCompass™ database for a one-year period from January 1st 2013 to December 31st 2013. Dogs ‘under veterinary care’ were defined as any dog with either at least one EPR recorded from January 1st to December 31st 2013 or, alternatively, at least one EPR both before and after 2013. Sample size calculations estimated that a sample of 149,282 dogs would be required to estimate an incidence risk for a disorder expected to occur in 1.0% of overall population with a 0.05% confidence limit assuming a UK population size of 8,000,000 dogs (Epi Info 7 CDC, 2019, Murray et al., 2010). Ethical approval was granted by the RVC Ethics and Welfare Committee (reference number SR2018–1652).

Case definition for an elbow joint disease case required that a final diagnosis of elbow joint disease (or synonym) was recorded in the EPR for a disorder that was present during the 2013 study period. The clinical decision-making process used for diagnosis of elbow joint disease was at the discretion of the attending veterinary surgeon. Case-finding involved initial screening of all EPRs for candidate elbow joint disease cases by searching the clinical free-text field and the VeNom term field using the single search term *elbow*. The candidate cases were randomly ordered and the clinical notes of a subset based on the power calculation estimate were manually reviewed in detail to evaluate for case inclusion. Information was extracted on cases to describe whether these were pre-existing (diagnosed prior to 2013) or incident (first recorded diagnosed during 2013) cases. Additional information extracted on the incident cases included the presenting signs described by the owners, whether the elbow joint disease was an incidental finding during a clinical examination for another presentation, specific type of elbow joint disease, findings recorded on veterinary examination, diagnostic process, medication, surgery and mortality information.

A *purebred* variable categorised all dogs of recognisable breeds as ‘purebred’ and the remaining dogs as ‘crossbred’ [39]. A *breed* variable included individual breeds represented by over 4000 dogs in the overall study population or with ≥ 7 incident elbow joint disease cases, a grouped category of all remaining purebreds and a general grouping of crossbred dogs. This approach was taken to facilitate statistical power for the individual breed analyses [40]. A *Kennel Club breed group* variable classified breeds recognised by the UK Kennel Club into their relevant breed groups (Gundog, Hound, Pastoral, Terrier, Toy, Utility and Working) and all remaining types were classified as non-Kennel Club recognised [39]. *Sex* (female, male, unavailable) and *neuter* (neutered, entire, unavailable) variables described the status recorded at the final EPR. An *insurance* variable described whether a dog was insured at any point during the study period. Age (years) was calculated for incident cases at the date of first recorded diagnosis and for all remaining dogs at the final date of the study period (December 31st, 2013). An *age* variable categorised age (years) into six groups (< 3.0, 3.0 - < 6.0, 6.0 - < 9.0, 9.0 - < 12.0, ≥ 12.0, unavailable). Adult bodyweight described the maximum bodyweight (kg) recorded at any date for dogs > 18 months old. An *adult bodyweight* variable categorised adult bodyweight into six groups (< 10.0 kg, 10.0 - < 20.0 kg, 20.0 - < 30.0 kg, 30.0 - < 40.0 kg, ≥ 40.0 kg, unavailable). A *bodyweight relative to breed mean* variable characterised the adult bodyweight of individual dogs as either below or equal/above the mean adult bodyweight for their breed and sex within the overall study population. This variable allowed assessment of adult bodyweight effects *within* each breed/sex combination.

Following data checking and cleaning in Excel (Microsoft Office Excel 2013, Microsoft Corp.), analyses were conducted using Stata Version 13 (Stata Corporation). The one-year period prevalence with 95% confidence intervals (CI) described the probability of evidence in the clinical records that confirmed the presence of elbow joint disease at any time during 2013. The elbow joint disease cases included both pre-existing (first diagnosed before 2013) and incident (newly diagnosed during 2013) cases. Because the sampling design involved manual verification of a subset of the candidate cases, the predicted case count for 2013 was calculated using the Stata *survey* function that weighted the verified case numbers by the inverse of the proportion of candidate cases manually confirmed [41]. The CI estimates were derived from standard errors, based on approximation to the binomial distribution [42]. This approach was repeated to similarly report the one-year incidence risk for newly diagnosed elbow joint disease cases during 2013. Descriptive statistics characterised the risk factors separately for the non-case and prevalent case dogs.

Risk factor analysis included only incident elbow joint disease dogs as cases while non-cases included all dogs that were not originally screened as candidate elbow joint disease cases. This focus on incident cases allowed interpretation of results as risk factors for “becoming” a case rather than for “being” a case [43]. Binary logistic regression modelling was used to evaluate univariable associations between risk factors (*purebred, breed, Kennel Club breed group, adult bodyweight, bodyweight relative to breed/sex mean, age, sex, neuter* and *insurance*) and elbow joint disease during 2013. Because breed was a factor of primary interest for the study, *purebred,* and *Kennel Club breed group* (variables that are highly collinear with breed) and *adult bodyweight* (a defining characteristic of individual breeds) were excluded from the initial breed multivariable modelling. Instead, each of these variables individually replaced the *breed* variable in the main final model in order to evaluate their effects after taking account of the other variables. Risk factors with liberal associations in univariable modelling (*P* <  0.2) were taken forward for multivariable evaluation. Model development used manual backwards stepwise elimination. Clinic attended was evaluated as a random effect and pair-wise interaction effects were evaluated for the final model variables [44]. The area under the ROC curve and the Hosmer-Lemeshow test were used to evaluate the quality of the model fit and discrimination (non-random effect model) [44, 45]. Statistical significance was set at *P* <  0.05.

## Results

### Demography

**Fig. 1:**
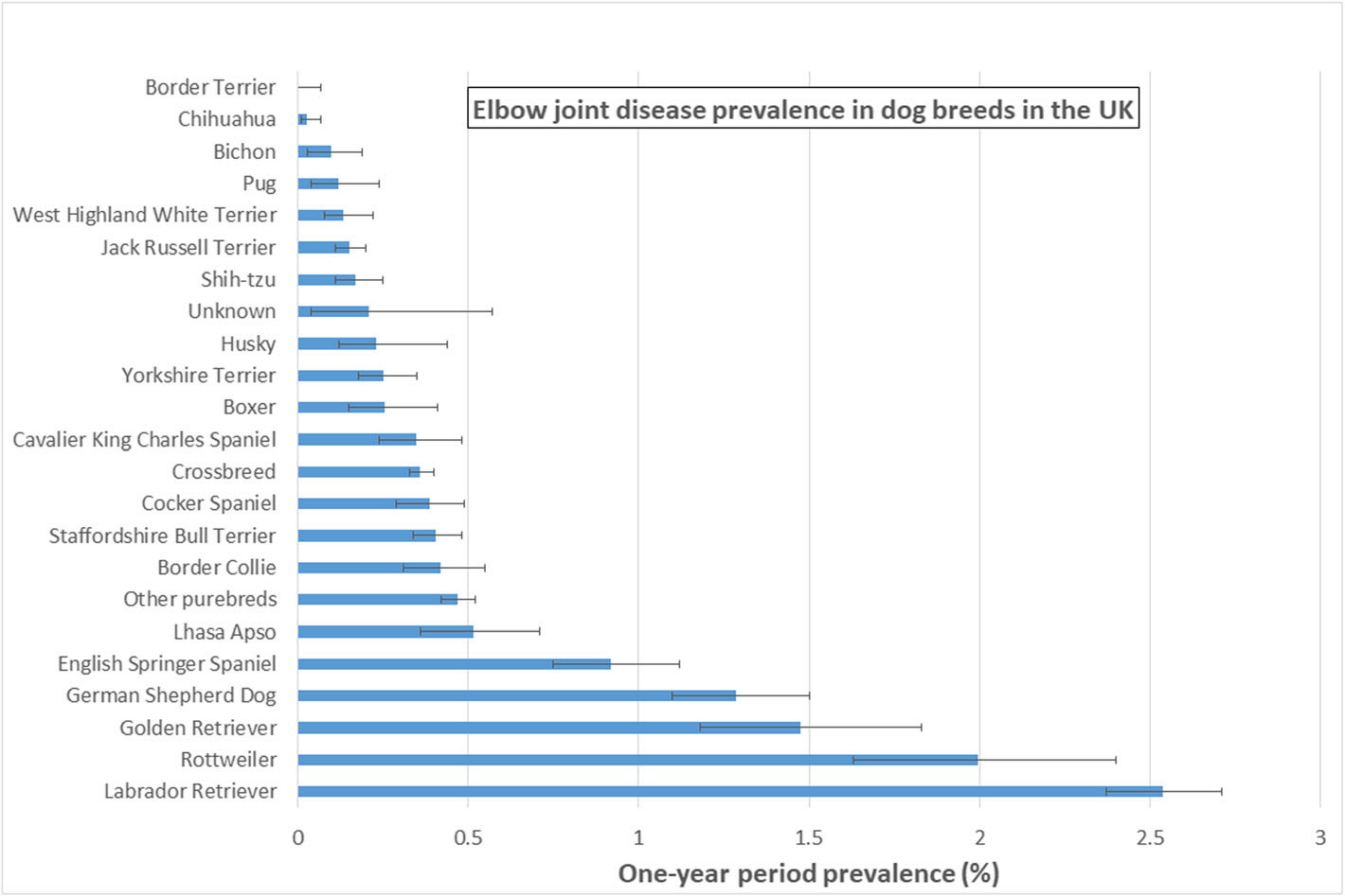
One-year (2013) period prevalence of elbow joint disease diagnosis in commonly affected dog breeds the VetCompass™ Programme under primary veterinary care in the UK in 2013. The error bars show the 95% confidence interval. (*n* = 804)

The denominator population comprised 455,069 dogs under veterinary care at 304 clinics in the UK during 2013. Of 12,060 candidate cases identified, 3751 (31.1%) were manually checked to confirm 804 elbow joint disease cases from this sample. After accounting for the effects of the subsampling protocol, the estimated one-year period prevalence for elbow joint disease diagnosis in dogs overall was 0.56% (95% CI: 0.53–0.60). The breeds with the highest elbow joint disease prevalence were Labrador Retriever (2.54, 95% CI 2.37–2.71), Rottweiler (1.99, 95% CI 1.63–2.40), Golden Retriever (1.47, 95% CI 1.18–1.83), German Shepherd Dog (1.28, 95% CI 1.10–1.50), and English Springer Spaniel (0.92, 95% CI 0.75–1.12) (Fig. 1). There were 616/804 (76.6%) of the overall cases that were incident in 2013. After accounting for the effects of the subsampling protocol, the estimated one-year incidence risk for elbow joint disease diagnosis was 0.45% (95% CI, 0.41–0.48) based on these 616 incident cases.

**Fig. 2:**
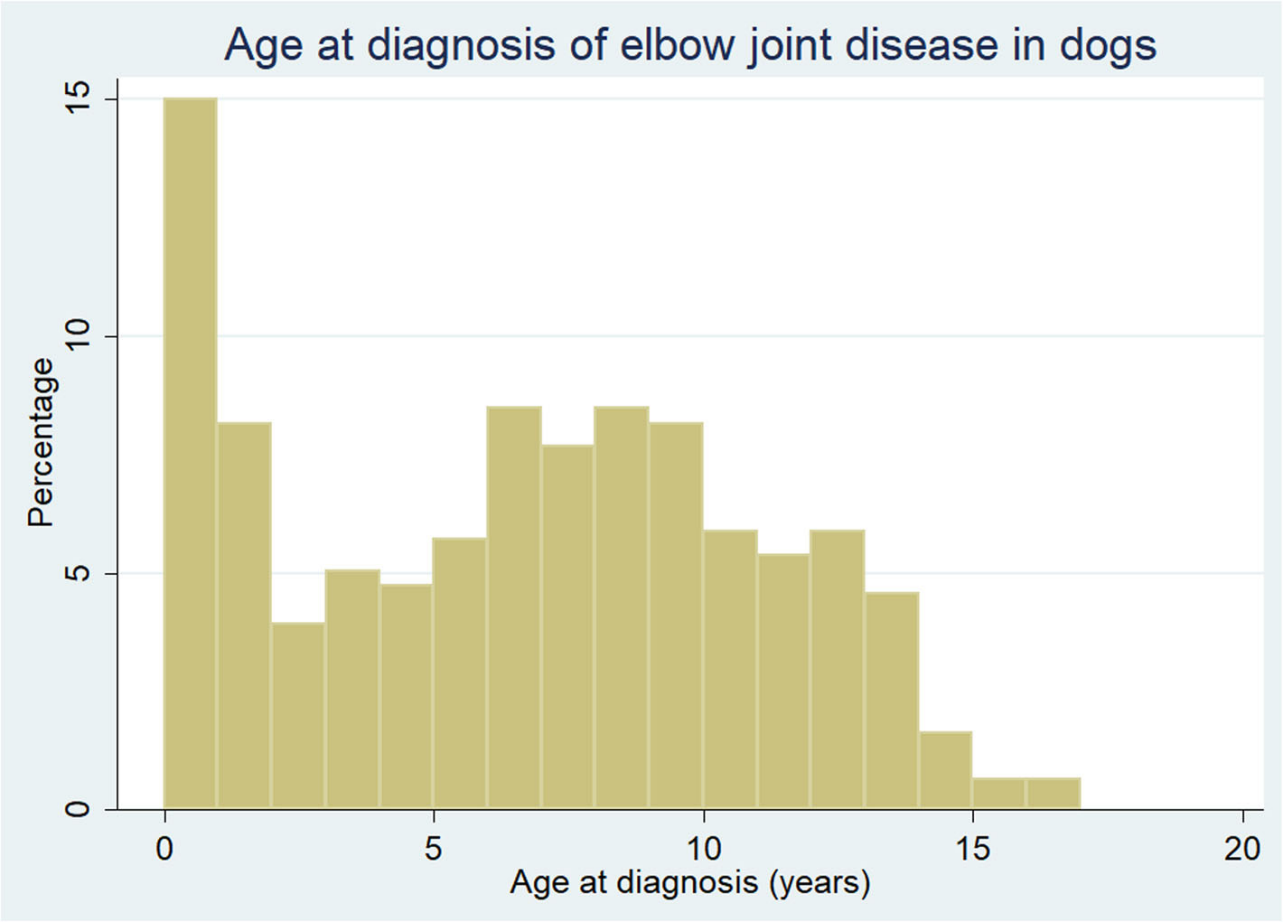
Distribution of age at first diagnosis of elbow joint disease diagnosis in dogs from the VetCompass™ Programme under primary veterinary care in the UK in 2013. (*n* = 616)

Of the incident elbow joint disease cases with data available for that variable, 515 (83.74%) were purebred, 244 (39.61%) were female, 360 (74.69%) were neutered and 182 (81.61%) were insured. Dogs with elbow joint disease had a median adult bodyweight of 32.45 kg (IQR: 22.00–40.00, range 3.00–88.00) and median age at diagnosis was 6.84 years (IQR: 2.45–9.97, range 0.23–17.00). The age distribution at diagnosis showed a bimodal pattern with a sharp peak in year 1 and a more gradual peak in years 6–10 (Fig. 2). The most common breeds among the incident elbow joint disease cases were Labrador Retriever (189, 30.68%), German Shepherd Dog (43, 6.98%), Staffordshire Bull Terrier (32, 5.19%) and Rottweiler (23, 3.73%), along with crossbred dogs (100, 16.23%) (Table 1). The median (IQR, count) age at first diagnosis for breeds with over 20 incident cases was: Labrador Retriever 6.42 years (2.50–9.08, *n* = 188), German Shepherd Dog 5.64 (0.80–7.77, 42), Staffordshire Bull Terrier 8.02 (3.16–10.87, 32), Rottweiler 7.20 (1.47–8.17, 23), English Springer Spaniel 7.00 (1.77–12.27, 21), Golden Retriever 9.75 (5.27–11.65, 21) and crossbred dogs 7.65 (3.39–10.95, 100).

**Table 1:** Descriptive and univariable logistic regression results for risk factors associated with incident diagnoses during 2013 of elbow joint disease in dogs under primary veterinary care in the VetCompass™ Programme in the UK. Column percentages shown in brackets. *CI confidence interval

Variable	Category	Case No. (%)	Non-case No. (%)	Odds ratio	95% CI*	Category *P*-value	Variable *P*-value
Purebred status	Crossbred	100 (16.26)	110,124 (24.96)	Base			< 0.001
	Purebred	515 (83.74)	331,113 (75.04)	1.72	1.38–2.12	< 0.001	
Breed	Crossbreed	100 (16.23)	110,124 (24.87)	1.00			< 0.001
	Labrador Retriever	189 (30.68)	30,894 (6.98)	6.74	5.29–8.59	< 0.001	
	Rottweiler	23 (3.73)	4999 (1.13)	5.07	3.22–7.98	< 0.001	
	Golden Retriever	21 (3.41)	5318 (1.20)	4.35	2.71–6.97	< 0.001	
	German Shepherd Dog	43 (6.98)	11,848 (2.68)	4.00	2.79–5.72	< 0.001	
	English Springer Spaniel	22 (3.57)	10,709 (2.42)	2.26	1.43–3.59	0.001	
	Other purebreds	100 (16.23)	82,952 (18.74)	1.33	1.01–1.75	0.045	
	Lhasa Apso	8 (1.30)	6773 (1.53)	1.30	0.63–2.67	0.474	
	Cavalier King Charles Spaniel	11 (1.79)	9924 (2.24)	1.22	0.65–2.28	0.53	
	Border Collie	13 (2.11)	11,834 (2.67)	1.21	0.68–2.16	0.519	
	Staffordshire Bull Terrier	32 (5.19)	31,897 (7.20)	1.10	0.74–1.65	0.624	
	Cocker Spaniel	14 (2.27)	15,458 (3.49)	1.00	0.57–1.75	0.993	
	Yorkshire Terrier	11 (1.79)	15,296 (3.45)	0.79	0.42–1.48	0.463	
	Unavailable	1 (0.16)	1502 (0.34)	0.73	0.10–5.26	0.758	
	Boxer	4 (0.65)	6075 (1.37)	0.73	0.27–1.97	0.529	
	Pug	2 (0.32)	5318 (1.20)	0.41	0.10–1.68	0.217	
	Jack Russell Terrier	10 (1.62)	27,407 (6.19)	0.40	0.21–0.77	0.006	
	West Highland White Terrier	4 (0.65)	11,838 (2.67)	0.37	0.14–1.01	0.053	
	Shih-tzu	5 (0.81)	14,882 (3.36)	0.37	0.15–0.91	0.03	
	Husky	1 (0.16)	4070 (0.92)	0.27	0.04–1.94	0.193	
	Bichon	1 (0.16)	6533 (1.48)	0.17	0.02–1.21	0.076	
	Chihuahua	1 (0.16)	11,709 (2.64)	0.09	0.01–0.67	0.019	
	Border Terrier	0 (0.00)	5379 (1.21)	~	~	~	
Kennel Club Breed Group	Breed not Kennel Club recognised	121 (19.67)	147,800 (33.50)	Base			< 0.001
	Toy	31 (5.04)	56,669 (12.84)	0.67	0.45–0.99	0.045	
	Utility	33 (5.37)	44,511 (10.09)	0.91	0.62–1.33	0.614	
	Terrier	49 (7.97)	57,913 (13.13)	1.03	0.74–1.44	0.846	
	Gundog	256 (41.63)	69,021 (15.64)	4.53	3.65–5.63	< 0.001	
	Hound	14 (2.28)	15,556 (3.53)	1.10	0.63–1.91	0.737	
	Pastoral	65 (10.57)	28,414 (6.44)	2.79	2.07–3.78	< 0.001	
	Working	46 (7.48)	21,353 (4.84)	2.63	1.87–3.70	< 0.001	
Adult (> 18 months) bodyweight (kg)	< 10.0	36 (5.84)	98,298 (22.20)	Base			< 0.001
	10.0 - < 20.0	89 (14.45)	89,741 (20.27)	2.71	1.84–3.99	< 0.001	
	20.0 - < 30.0	115 (18.67)	65,740 (14.85)	4.78	3.28–6.95	< 0.001	
	30.0 - < 40.0	187 (30.36)	43,560 (9.84)	11.72	8.20–16.75	< 0.001	
	≥ 40.0	147 (23.86)	23,153 (5.23)	17.34	12.04–24.97	< 0.001	
	Unavailable	42 (6.82)	122,247 (27.61)	0.94	0.60–1.46	0.779	
Bodyweight relative to breed mean	Lower	200 (32.47)	179,134 (40.46)	Base			< 0.001
	Equal/Higher	374 (60.71)	140,861 (31.82)	2.38	2.00–2.82	< 0.001	
	Unavailable	42 (6.82)	122,744 (27.72)	0.31	0.22–0.43	< 0.001	
Age (years)	< 3.0 years	108 (17.53)	173,638 (39.22)	Base			< 0.001
	3.0 - < 6.0 years	103 (16.72)	112,371 (25.38)	1.47	1.12–1.93	0.005	
	6.0 - < 9.0 years	142 (23.05)	74,082 (16.73)	3.08	2.40–3.96	< 0.001	
	9.0 - < 12.0 years	149 (24.19)	44,891 (10.14)	5.34	4.16–6.84	< 0.001	
	≥ 12.0 years	112 (18.18)	31,666 (7.15)	5.69	4.36–7.41	< 0.001	
	Unavailable	2 (0.32)	6091 (1.38)	0.53	0.13–2.14	0.371	
Sex	Female	244 (39.61)	213,489 (48.22)	Base			< 0.001
	Male	372 (60.39)	226,969 (51.26)	1.43	1.22–1.69	< 0.001	
	Unavailable	0 (0.00)	2281 (0.52)	~	~	~	
Neuter	Entire	122 (19.81)	174,753 (39.47)	Base			< 0.001
	Neutered	360 (58.44)	198,298 (44.79)	2.60	2.12–3.19	< 0.001	
	Unavailable	134 (21.75)	69,688 (15.74)	2.75	2.15–3.52	< 0.001	
Insurance	Non-insured	27,320 (6.17)	41 (6.66)	Base			< 0.001
	Insured	54,136 (12.23)	182 (29.55)	2.24	1.60–3.14	< 0.001	
	Unavailable	361,283 (81.60)	393 (63.80)	0.72	0.53–1.00	0.050	

Of the non-case dogs with data available on the variable, 331,113 (75.04%) were purebred, 213,489 (48.47%) were female, 198,298 (53.16%) were neutered and 54,136 (66.46%) were insured. The median adult bodyweight for non-cases was 16.20 kg (IQR: 8.90–27.50, range 0.30–99.95) and the median age was 4.06 years (IQR: 1.69–7.52, range 0.00–24.68). The most common breeds among the non-case dogs were Staffordshire Bull Terrier (31,897, 7.20%), Labrador Retriever (30,894, 6.98%), Jack Russell Terrier (27,407, 6.19%) and Cocker Spaniel (15,458, 3.49%) accompanied by a substantial population of crossbred dogs (110,124, 24.87%) (Table 1). Data completeness varied between the variables assessed: breed 99.66%, age 98.65%, sex 99.50%, bodyweight at any age 88.94%, insurance 18.78%, and neuter 84.12%. There were 376 (61.04%) cases recorded as bilateral with 123 (19.97%) affected only on the left side and 117 (18.99%) affected only on the right side.

### Clinical care

Of the 616 incident cases, the following proportions of specific types of elbow joint disease were recorded at any date in the clinical records: osteoarthritis (or degenerative joint disease) (*n* = 468, 75.97%), elbow dysplasia (190, 30.84%), traumatic (41, 6.66%), septic arthritis (11, 1.79%), immune mediated (7, 1.14%), polyarthritis (3, 0.49%) and neoplastic (3, 0.49%). The most common presenting signs recorded as described by the owners were lameness (*n* = 466, 75.65%), difficulty exercising (123, 19.97%), pain (86, 13.96%) and inflammation (4, 0.65%).

The elbow joint disease was noted as an incidental finding during a clinical examination for presentation for another reason in 63 (10.23%) cases. The most common findings recorded on veterinary examination were pain (*n* = 283, 45.94%), lameness (278, 45.13%), reduced range of movement (243, 39.45%), crepitus (162, 26.30%) and inflammation or joint thickening (133, 21.59%). The diagnostic process included the following aids: radiography (*n* = 330, 53.57%), computerized tomography (37, 6.01%), arthroscopy (20, 3.26%) and magnetic resonance imaging [MRI] (2, 0.33%). Among 330 dogs that had at least one of these four diagnostic procedures, the most common specific diagnoses recorded were osteoarthritis (*n* = 101, 30.61%) and coronoid disease (81, 24.55%) (Table 2). Overall, 153 (24.84%) of the elbow joint cases were referred for advanced veterinary care.

**Table 2:** Specific veterinary diagnoses recorded in dogs under primary veterinary care in the VetCompass™ Programme in the UK with elbow joint disease that had at least one of the following: radiography, computerized tomography [CT], arthroscopy and magnetic resonance imaging [MRI]. *N* = 330

Diagnosis	Frequency	Percent
Osteoarthritis	101	30.61
Coronoid disease	81	24.55
Inconclusive	54	16.36
Fracture	26	7.88
Elbow incongruity	25	7.58
Osteochondritis dissecans	19	5.76
Ununited anconeal process	11	3.33
Incomplete ossification of the humeral condyle	7	2.12
Joint mouse	2	0.61
Neoplasia	2	0.61
Congenital elbow luxation	1	0.30
Polyarthritic elbow joint disease	1	0.30
Total	330	

At least one medication was prescribed or used on 573 (93.02%) cases. Common medications used included non-steroidal anti-inflammatory drugs [NSAIDS] (*n* = 544, 88.31%), tramadol (121, 19.64%), disease modifying agents (118, 19.16%), systemic glucocorticoids (19, 3.08%), gabapentin (11, 1.79%), intra-articular medications (5, 0.81%), amantadine (3, 0.49%). Nutraceuticals were used or recommended in 248 (40.26%) of cases. Therapeutic surgery was performed on 85 (13.80%) cases.

Of 167 incident cases that had died by the end of the study, the median age at death was 12.09 years (IQR 9.69–13.90, range 0.94–17.10). Of 153 dogs with information recorded, 146 (95.42%) deaths involved euthanasia while 7 (4.58%) were unassisted. Of 109 euthanasia cases with information available, elbow joint disease contributed to the decision to euthanase in 45 (41.28%) dogs.

### Risk factors

All tested variables were liberally associated with elbow joint disease in univariable logistic regression modelling and were evaluated using multivariable logistic regression modelling as described in the methods (Table 1). The final main breed-focused multivariable model retained six risk factors: *breed, bodyweight relative to breed-sex mean*, *age, sex, neutered* and *insurance* (Table 3). No biologically significant interactions were identified. The final model was improved by inclusion of the clinic attended as a random effect (rho: 0.03 indicating that 3% of the variability was accounted for by the clinic attended, *P* <  0.001) and these results were reported. The final unclustered model showed acceptable model-fit (Hosmer-Lemeshow test statistic: *P* = 0.089) and good discrimination (area under the ROC curve: 0.829).

**Table 3:** Final breed-focused mixed effects multivariable logistic regression model for risk factors associated with diagnosis of elbow joint disease in dogs under primary veterinary care in the VetCompass™ Programme in the UK. *CI confidence interval

Variable	Category	Odds ratio	95% CI*	*P*-value
Breed	Crossbreed	1.00		
	Rottweiler	6.16	3.89–9.75	< 0.001
	Labrador Retriever	5.94	4.65–7.60	< 0.001
	German Shepherd Dog	4.13	2.88–5.93	< 0.001
	Golden Retriever	3.11	1.93–5.00	< 0.001
	English Springer Spaniel	2.00	1.26–3.18	0.003
	Other purebreds	1.39	1.05–1.84	0.021
	Lhasa Apso	1.37	0.66–2.82	0.396
	Unknown	1.36	0.19–9.79	0.761
	Staffordshire Bull Terrier	1.27	0.85–1.89	0.243
	Cavalier King Charles Spaniel	1.15	0.62–2.15	0.655
	Border Collie	1.04	0.58–1.85	0.900
	Cocker Spaniel	0.95	0.54–1.67	0.859
	Yorkshire Terrier	0.74	0.40–1.39	0.353
	Boxer	0.68	0.25–1.85	0.452
	Pug	0.66	0.16–2.67	0.557
	Shih-tzu	0.46	0.19–1.14	0.095
	Husky	0.41	0.06–2.96	0.379
	Jack Russell Terrier	0.38	0.20–0.72	0.003
	West Highland White Terrier	0.27	0.10–0.73	0.010
	Bichon	0.18	0.03–1.30	0.090
	Chihuahua	0.16	0.02–1.18	0.072
	Border Terrier	0.00	~	0.991
Bodyweight relative to breed/sex mean	Lower	1.00		
	Equal/Higher	2.00	1.68–2.39	< 0.001
	Unavailable	0.48	0.34–0.67	< 0.001
Age (years)	< 3.0	1.00		
	3.0 - < 6.0 years	0.84	0.64–1.11	0.223
	6.0 - < 9.0 years	1.47	1.13–1.92	0.004
	9.0 - < 12.0 years	2.56	1.97–3.32	< 0.001
	> or = 12.0 years	3.70	2.81–4.87	< 0.001
	Unavailable	0.80	0.20–3.26	0.758
Sex	Female	1.00		
	Male	1.47	1.25–1.73	< 0.001
	Unavailable	0.00	~	0.995
Neutered	Entire	1.00		
	Neutered	1.69	1.37–2.10	< 0.001
	Unavailable	1.54	1.16–2.05	0.003
Insurance	Uninsured	1.00		
	Insured	2.32	1.64–3.29	< 0.001
	Unavailable	1.17	0.82–1.67	0.381

After accounting for the effects of the other variables evaluated, five breeds showed increased odds of elbow joint disease compared with crossbred dogs: Rottweiler (OR: 6.16, 95% CI 3.89–9.75, *P* <  0.001), Labrador Retriever (OR: 5.94, 95% CI 4.65–7.60, *P* <  0.001), German Shepherd Dog (OR: 4.13, 95% CI 2.88–5.93, *P* <  0.001), Golden Retriever (OR: 3.11, 95% CI 1.93–5.00, *P* <  0.001) and English Springer Spaniel (OR: 2.00, 95% CI 1.26–3.18, *P* = 0.003). Two breeds showed reduced odds of elbow joint disease compared with crossbreds: Jack Russell Terrier (OR: 0.38, 95% CI 0.20–0.72, *P* = 0.003) and West Highland White Terrier (OR: 0.27, 95% CI 0.10–0.73, *P* = 0.010). Individual dogs with an adult bodyweight that was equal or higher than their breed/sex mean had 2.00 (95% CI 1.68–2.39, *P* <  0.001) times the odds of elbow joint disease compared with dogs that weighed below their breed/sex mean. Ageing was associated with increasing odds of elbow joint disease. Compared with dogs aged < 3.0 years, dogs aged 9.0 - < 12.0 years had 2.56 times the odds (95% CI 1.97–3.32, *P* <  0.001) of elbow joint disease. Males had 1.47 times the odds (95% CI 1.25–1.73, *P* <  0.001) of elbow joint disease compared with females. Neutered animals had 1.69 times the odds (95% CI 1.37–2.10, *P* <  0.001) of elbow joint disease compared with entire animals. Insured dogs had 2.32 (95% CI 1.64–3.29, *P* <  0.001) times the odds of elbow joint disease compared with uninsured dogs (Table 3).

As described in the methods, *purebred* and *Kennel Club breed group* individually replaced the *breed* variable in the final breed-focused multivariable model while *adult bodyweight* replaced *breed* and *bodyweight relative to breed/sex mean*. Purebred dogs had 1.70 times the odds (95% CI 1.37–2.10, *P* <  0.001) compared with crossbred dogs. Three of the seven Kennel Club breed groups showed higher odds of elbow joint disease compared with dogs of breeds that are not recognized by the Kennel Club: Gundog (OR: 3.94, 95% CI 3.17–4.90, *P* <  0.001), Working (OR: 3.00, 95% CI 2.13–4.23, *P* <  0.001) and Pastoral (OR: 2.54, 95% CI 1.87–3.43, *P* <  0.001). The odds of elbow joint disease increased substantially as adult bodyweight increased. Dogs weighing 30.0- < 40.0 kg had 9.84 times the odds (95% CI: 6.87–14.08, P <  0.001) (Table 4).

**Table 4:** Results for Purebred status and Kennel Club Breed Group after replacing the breed variable in the final breed-focused mixed effects multivariable logistic regression model (along with age, bodyweight relative to breed mean, sex, neutered and insurance status) and for Adult (> 18 months) bodyweight (kg) that replaced the breed and bodyweight relative to breed mean variables in the final breed-focused mixed effects multivariable logistic regression model (with age, sex, neutered and insurance status). These results report associations between these risk factors and a diagnosis of elbow joint disease in dogs under primary veterinary care in the VetCompass™ Programme in the UK. *CI confidence interval

Variable	Category	Odds ratio	95% CI*	Category *P*-value
Purebred status	Crossbred	1.00		
	Purebred	1.70	1.37–2.10	< 0.001
Kennel Club Breed Group	Breed not KC-recognised	1.00		
	Gundog	3.94	3.17–4.90	< 0.001
	Working	3.00	2.13–4.23	< 0.001
	Pastoral	2.54	1.87–3.43	< 0.001
	Hound	1.06	0.61–1.84	0.843
	Utility	1.04	0.71–1.53	0.851
	Terrier	0.98	0.70–1.37	0.921
	Toy	0.76	0.51–1.12	0.164
Adult (> 18 months) bodyweight (kg)	< 10.0	1.00		
	10.0 - < 20.0	2.35	1.59–3.46	< 0.001
	20.0 - < 30.0	4.10	2.81–5.96	< 0.001
	30.0 - < 40.0	9.84	6.87–14.08	< 0.001
	≥ 40.0	15.03	10.39–21.74	< 0.001
	Unavailable	1.33	0.84–2.10	0.218

## Discussion

To date, this is the largest primary-care veterinary study to provide epidemiological data on elbow joint disease in dogs and revealed a significant burden of elbow osteoarthritis in the wider dog population. Reliable prevalence data that is relevant to primary care is needed to help inform and focus health reforms in dogs, particularly those associated with breed characteristics [46, 47]. A prevalence of 0.56% was shown from a population of 455,069 dogs from 304 clinics, indicating elbow joint disease is not as prevalent as patellar luxation [48] but had a similar prevalence to cruciate ligament rupture which is considered a significant health and financial burden [49]. Although previous estimates of breed prevalence for elbow dysplasia has varied from 0.01 to 0.89 [50], these populations are usually based on screening programmes and therefore do not represent the wider true dog population. Studies based on data derived from international schemes for assessing hip and elbow dyplasia are often biased through positive selection of dogs with phenotypically ‘good’ joints [51], the voluntary nature of these schemes, the relatively low uptake across the total population of dogs, and the inclusion of predisposed phenotypes. Even after 40 years of enrolment in some programmes, there has been only minor changes reported in elbow dysplasia disease prevalence [52]. The application of anonymised primary care veterinary data which have not been ‘vetted’ in advance of inclusion in analyses and that are not biased to breeds which are known to have elbow dysplasia (and hence subject to screening) are therefore more likely to capture a representative estimate of disease for the entire population of dogs. Of course, these data rely heavily on the accuracy and completeness of clinical records which can be affected by differing diagnostic options and variable data recording of a range of clinical conditions.

The current study substantiated some previously reported breed-related variation in prevalence of elbow disease. The breeds with the highest prevalence were mainly large breeds and included Labrador Retriever, Rottweiler, Golden Retriever, German Shepherd Dogs, and English Springer Spaniels. Additional breeds with high prevalence of elbow dysplasia based on data from screening programmes, but not identified from the current primary care study included the Chow Chow, Bernese Mountain Dog and Newfoundland [50]. Male and neutered dogs were also more prevalent in the elbow disease group, with around 60% being male and 75% neutered. A predominance of male dogs has been reported previously for medial coronoid disease (the commonest form of elbow dysplasia) [1], and the male to female ratio for elbow dysplasia in Labradors and Golden Retrievers has been shown to be 2.2:1 [21, 22, 53]. The predisposition of male dogs could be due to dominant inheritance with reduced penetrance in female dogs, or associated factors such as neutering, exercise levels, growth rates and overall weight [54].

Purebred dogs had 1.7 times the odds of diagnosis of elbow disease, and the breeds at greatest risk of elbow disease included some of those previously identified as at risk of osteoarthritis and elbow dysplasia, in addition to English Springer Spaniels. The breeds with the highest risk of elbow disease were Labrador Retriever, Rottweiler, Golden Retriever, and German Shepherd, and these breeds have also previously demonstrated increased risk for osteoarthritis development [30]. Therefore, the elbow may be a significant contributing joint to the overall levels of osteoarthritis seen in the dog population. The breed-specific risk and increased prevalence in purebreds are suggestive of a genetic component to elbow disease and this is corroborated by heritability studies. Labrador Retriever heritability in the UK for elbow dysplasia was reported at 0.19 based upon 3613 elbow scores [25]. German Rottweilers have been reported with heritability of 0.28 [22], and Swedish Rottweilers at 0.34 [24]. German Shepherds have a heritability for elbow dysplasia of 0.6 [55], and 0.45 for Golden Retrievers [53]. The identification of increased risk in English Springer Spaniels in the current study may be due to their breed association with humeral intracondylar fissures (HIFs), which can predispose to lameness and low energy fractures [5, 6]. In a prospective observational study of English Springer Spaniels without a history of lameness, CT scans revealed HIF in 14% of the dogs, and around 50% had medial coronoid process disease changes, with a total of 60% of apparently clinically normal dogs showing osteoarthritic change [56]*.* Data from the subset of dogs having diagnostic imaging in this study identified IOHC/HIF in 2% of these dogs. It is interesting that the groups of dogs bred for working, including Kennel Club Gundog, Pastoral and Working groups, all had significantly increased odds ratios for elbow disease. Whether elbow disease manifests more in these dogs due to their breed intended growth rates, sizes or elbow conformation remains unclear at this stage.

This study also identified breeds protected for elbow disease, including Jack Russell Terriers and West Highland White Terriers. These were not the same breeds as previously reported at reduced risk in the most up-to-date long-term analysis of screening programme data which included 500 dogs. That study identified Boxer, Flat coated retriever, Bichon, Cavalier King Charles spaniel and Briard as low risk for elbow dysplasia [50]. The current study identified Boxers with an odds ratio of 0.68, although Cavalier King Charles spaniels had an odds ratio of 1.15. This difference could relate to the current study being more inclusive of elbow disease in general, or simply because it represents a bigger sample population which is not biased by owner decision-making on which dogs are submitted for radiographic survey.

In this current large population study, 61% of cases were diagnosed with bilateral elbow disease. The current study covered all types of elbow disease, whereas most prior information determining rates of bilateralism comes from elbow dysplasia studies with 25–80% of dogs reported with bilateral disease [8, 57, 58]. In any case, the high level of bilateral disease and functional impairment suggests that elbow joint disease is a significant welfare problem for affected individuals.

The differences identified here between owner perception and veterinary assessments are worth considering in terms of welfare and public education. Owners predominantly focussed on externally visible clinical signs such as lameness (75.65%) or problems exercising (19.97%), with only a smaller proportion reporting internal affective issues such as pain (13.96%) as a presenting complaint. Conversely, veterinary assessment recorded pain (45.94%) as the most common clinical finding. Dog owners have previously been shown to be poor at predicting and interpretting behavioural adaptation to pain [59], and therefore reliance on dog owners to identify chronic pain may be unsafe [60]. Additionally, 10.23% of elbow joint disease cases were identified incidentally at routine veterinary appointments which suggests that many owners may normalise these clinical signs as typical of aging. Veterinary examination can reveal and detect aspects of joint disease that are not appreciable to untrained owners and it may be unfair to assume that all elbow joint cases should be easily recognisable to owners. Typical clinical signs indicative of advanced joint remodelling associated with osteoarthritis were identified on veterinary examination have been reported in 1/5 (thickening) to 1/4 (crepitus) dogs, and 50% of diagnoses were made on clinical examination [61].

Consistent with the predominance of older dogs identified with elbow joint disease in the current study, osteoarthritis (degenerative joint disease) was diagnosed in over 75% of cases. As discussed above, these later stage presentations are most likely secondary to pre-existing elbow dysplasia but could also result from some forms of primary osteoarthritis [16]. Studies show that both conservative management [62] and surgical treatment of elbow dysplasia ultimately lead to osteoarthritis [63, 64]. The protracted time lag from inciting cause to clinical presentation for chronic diseases such as osteoarthritis combined with the data-availability constraints of our type of epidemiological study did not permit fuller exploration of the natural history of these cases. It does however clearly highlight that, by whatever the route, elbow osteoarthritis is the most common disease present in the elbows of dogs under primary care, with its substantial impact on pain, mobility and welfare. From a welfare perspective, it is further notable that their elbow joint disease, which was mostly diagnosed as osteoarthritis, contributed to the decision to euthanase in 41.28% of the cases euthanased during the study, indicating another significant welfare impact. An exploration of summary welfare impact among common disorders of dogs in the UK identified osteoarthritis with the second highest overall welfare impact score among the eight disorders evaluated [65]. The current study also provides the first indication of a population incidence for rare elbow joint diseases such as septic or immune mediated polyarthritis, both being under 2%, and corroborates the limited literature from a handful of small case series from referral populations [2, 66–68].

A tendency towards increased diagnosis in insured dogs was shown here and has also been demonstrated for other orthopaedic conditions [30, 48, 69]. Reduced financial restrictions or potentially differing client/owner expectations when insured may underlie this recurring phenomenon. Interestingly, the influence of insurance on diagnostic outcomes appears to vary by condition, being highest for cruciate ligament rupture (4x), then elbow disease (2.35x) and then patellar luxation (1.9x). This perhaps reflects the differential confidence by primary-care practitioners in diagnosis-making between these conditions and the subsequent need for additional costly diagnostics to assess the more uncertain diagnoses. In the current study, only around half of cases included diagnostic imaging within diagnostic protocols used, suggesting a relatively high level of clinical confidence in these diagnoses being made in primary veterinary care. Plain radiography dominated among the diagnoses that included diagnostic imaging, perhaps due to its ease of use and fairly universal access in primary care. However, plain radiography is associated with a relatively high proportion of false-negative diagnoses (10–69%) in large groups of dogs with elbow related lameness [70, 71]. Hence, advanced imaging is frequently recommended but often requires referral to ensure access to this diagnostic modality. Direct diagnosis of common forms of elbow dysplasia (coronoid disease, elbow incongruity, osteochondritis dissecans and ununited anconeal process) accounted for 41% of diagnoses, with medial coronoid process disease accounting for 59% of elbow dysplasia types. This is lower than reported in screening programme populations [72] with medial coronoid disease being present in 96% of elbow dysplasia dogs, perhaps because the use of plain radiography significantly reduces accurate diagnosis [70, 71].

Despite many years of research, the exact aetiology of elbow dysplasia remains unclear. Several overarching theories have been described including osteochondrosis [73], differing types of elbow incongruity [54], and muscular biomechanical force mismatch [74]. Whatever the aetiology, these disease entities are considered to be the result of the interplay of genetics and environment such as high energy diets driving rapid growth or excessive exercise [4]. Although elbow dysplasia and joint incongruity have been well described as a driver of joint arthrosis in young dogs [1, 12, 75], osteoarthritis development in absence of a primary alternative joint disease, so called primary osteoarthritis, is thought to be rare [11].

Being above average bodyweight for the breed and sex was identified as a significant risk factor for elbow disease. Experimental dog colonies have clearly demonstrated that increased calorie intake and hence increased bodyweight positively are associated with increased levels of osteoarthritis. In a longitudinal study following seven litters of Labrador Retrievers where one group was fed ad-lib and the other 25% fewer calories, the reduced-calorie group had 26% lower mean body mass. The ad-lib fed dogs showed significantly greater radiographic severity of osteoarthritic change at 6 years of age, although histopathology did not any differences at end of life. Overall, by 6 years of age, radiographic osteoarthritis was seen in 19.1% of dogs [16]. Age was significantly associated with prevalence and severity of osteoarthritis in the Labrador Retriever colony dogs, which was also demonstrated as a risk factor for incidence in the current study. Elbow dysplasia has historically been considered one of the main causes of elbow osteoarthritis [1, 11, 12, 75], however, rather unexpectedly, none of the dogs in the colony had any indication of a pre-existing disease such as elbow dysplasia based on the radiographic and histological signs of the presence of a fragmented medial coronoid process (FMCP), un-united anconeal process (UAP) and osteochondrosis or osteochondritis dissecans (OCD). For the first time, elbow osteoarthritis was documented as a potentially primary osteoarthritis. This suggests that some of the dogs identified in the current study with elbow disease may have had primary osteoarthritis.

The clinical management of elbow joint disease in the current study, dominated by osteoarthritis, included at least one medication in most dogs. Medication included a predominance of NSAIDs (88%) and also a relatively high level of tramadol usage (19%). Tramadol for osteoarthritis management has been popularised in recent years following concerns over side-effects from NSAIDs [76]. However a recent randomised placebo controlled, cross-over, double blinded study irrefutably demonstrated that tramadol was only as effective as placebo and was significantly inferior to NSAIDs [77]. Interestingly, when compared to management of osteoarthritis overall in dogs, medical treatment is prescribed more frequently for the elbow than for osteoarthritis in general (93% vs 75% respectively) [30]. This may reflect more debilitating effects perceived for osteoarthritis in the elbow compared with some other joints. Nutraceuticals were commonly used in primary-care practice, with 40% of dogs receiving them. Some elbow joint disease dogs were treated by surgical intervention in primary veterinary care (14%), presumably for a primary disease such as medial coronoid process disease.

Two age related peaks in incidence were seen, corroborating the young and the old patterns previously described in referral populations [4]. In those referral populations, this pattern was attributed to primary elbow dysplasia driving lameness when young whereas the later peak has been attributed to the subsequent secondary osteoarthritis [1]. A risk factor for osteoarthritis is increasing age, notably above 8 years [30], and a similar peak was demonstrated for elbow disease with a peak in the 8–10 year range. When arthroscopic findings from 600 dogs with elbow lameness were reviewed, nearly 50% were in the younger age group peak (5-18 months of age), and 12% were in an old dog group (> 6 years). There was a notable difference in pathology with medial compartment erosions (deep ulcerations of the medial part of the joint with exposure of the subchondral bone – Modified Outerbridge Score 4) in 31% of old dogs and only 3% of young dogs. The Bernese Mountain dog was not seen in the older group, whereas mixed breeds were over-represented [78], suggestive perhaps of different disease entities occurring in the elbow of older vs young dogs, although in both groups medial coronoid disease predominated.

The limitations of using primary-care veterinary clinical data for research have been previously published [27, 33, 79]. The current study was limited by its retrospective nature and the use of clinical data that were not recorded primarily for research purposes and which therefore may have allowed some disease status misclassification. This study may have underrepresented elbow joint disease because true cases in the denominator population that were not presented for veterinary care during 2013 were not included as cases. It is also difficult to distinguish the natural history of elbow disease, as we can only presume elbow dysplasia to be a common underlying cause of the high level of elbow osteoarthritis. The bimodal incidence distribution, with a young dog peak in the first 2 years of life and a broader old dog peak fits with the literature descriptions of elbow dysplasia in the young leading to secondary osteoarthritis in the older dog. Furthermore, similar sex and breed distributions that are described for elbow dysplasia were also seen in the current study [22, 24, 25, 53, 55]. Although the current study could not determine the proportion of osteoarthritis cases that had underlying elbow dysplasia, the study clearly highlights that, at any one time, there is a large population of dogs with elbow osteoarthritis in primary veterinary practice. This study also gives the most accurate measure of the prevalence of elbow disease in primary care dogs, and also provides, for the first time, an index of prevalence for rare conditions such as septic and immune mediated elbow joint disease. This study excluded dogs that were not under veterinary care and therefore may have introduced bias toward the increasingly neutered, insured and more closely monitored subset of the population that do receive veterinary care. Body condition scores were not available for this study and therefore analysis of association between obesity and elbow joint disease, although desirable, was not possible.

## Conclusions

This is the largest epidemiological study based on primary care veterinary data to evaluate elbow joint disease in dogs and shows a prevalence of 0.56% in the UK. There were strong breed predispositions, in particular for large breed dogs, such as Labrador, Rottweiler, Golden Retriever and German Shepherd Dog, which align to breeds shown to be over-represented in elbow dysplasia studies. Notably, there was a very high level of bilateral disease at 61%, and being male, neutered and weighing above the breed average were significant risk factors. Osteoarthritis was by far the most common specific cause for elbow joint disease. Tramadol is frequently used to manage the disease, although recent evidence worryingly suggests this is no more effective than placebo [80]. This study identified a significant welfare burden from elbow joint disease with over 40% of euthanasia cases during that period being attributed to their elbow joint disease. Based on the breed predisposition, high level of bilateral disease and impact on welfare, there is a significant case for improving breeding programmes and developing improved genetic assessment tools to reduce the burden of elbow joint disease.

## Data Availability

The VetCompass dataset used for this study are available open access on the RVC data repository: http://researchonline.rvc.ac.uk/id/eprint/12408/ .

## Abbreviations

CI: Confidence interval
CT: Computerized tomography
EPR: Electronic patient record
FMCP: Fragmented medial coronoid process
HIF: Humeral intracondylar fissure
IEWG: The International Elbow Working Group
IQR: Interquartile range
KC: The Kennel Club
MRI: Magnetic resonance imaging
NSAIDS: Non-steroidal anti-inflammatory drugs
OCD: Osteochondritis dissecans
OR: Odds ratio
UAP: Ununited anconeal process
